# Experimentally measured assembly indices are required to determine the threshold for life

**DOI:** 10.1098/rsif.2024.0367

**Published:** 2024-11-20

**Authors:** Sara I. Walker, Cole Mathis, Stuart Marshall, Leroy Cronin

**Affiliations:** ^1^ School of Earth and Space Exploration, Arizona State University, Tempe, AZ 85287-0506, USA; ^2^ School for Complex Adaptive Systems, Arizona State University, Tempe, AZ 85287-0506, USA; ^3^ School of Chemistry, The University of Glasgow, Glasgow, UK

**Keywords:** assembly theory, assembly indices, evolution, selection, mineral complexity

## Abstract

Assembly theory (AT) aims to distinguish living from non-living systems by explaining and quantifying selection and evolution. The theory proposes that the degree of assembly depends on the number of complex objects, with complexity measured using a combination of the object’s assembly index (AI) and its abundance. We previously provided experimental evidence supporting AT’s predictive power, finding that abiotic systems do not randomly produce organic molecules with an AI greater than approximately 15 in detectable amounts. Hazen *et al*. (Hazen *et al*. 2024 *J. R. Soc. Interface* **21**, 20230632. (doi:10.1098/rsif.2023.0632)) proposed inorganic molecules that theoretically have AIs greater than 15, suggesting similar complexity to biological molecules. However, our AIs are experimentally measured for organic, covalently bonded molecules, whereas Hazen’s are theoretical, derived from crystal structures of charged units that are not isolable in solution. This distinction underscores the challenge in experimentally validating theoretical AIs.

*J. R. Soc. Interface*; **21**: 20240622 (Published online 20 November 2024). (https:// doi.org/10.1098/rsif.2024.0622)

One key suggestion relates to doing calculations of AI on heteropolyanions, while also considering what experimental data might be useful to measure the AIs of the materials. Before we do this, it is useful to reiterate the fundamental premise of AT. This is because AT introduces the concept of an object (such as a molecule) as an entity that is finite, distinguishable, persists in time and is breakable such that the set of constraints to construct it is quantifiable [4]. Thus, the complexity of an object is quantified by defining an intrinsic measure called AI ($a_{i}$), which is the shortest number of recursive steps to construct an object. For a collection of objects, we have defined an integrated quantity assembly (A), which quantifies the total amount of selection necessary to produce a set of observed objects with two parameters, as quantified using equation (1.1)


(1.1) $$A=\sum_{i=1}^{N}e^{a_{i}}\;\left(\frac{n_{i}-1}{N_{T}}\right),$$

where $a_{i}$ is the AI of ith object, $n_{i}$ is its copy number or concentration and $N_{T}$ is the total number of objects in the sample [4]. Thus, the combination of AI and copy number quantifies, within the large combinatorial space, how a selection process led to the discovery of new objects and, among them, the system’s capacity to produce a high copy number of specific objects. However, the meaning of the assembly is only valid as a physical quantity if both the AI and copy number, i.e. abundance, can be determined experimentally.

We define the shortest pathway to construct the object as the *assembly pathway* on which the AI (also called molecular assembly for molecules) has been quantified. In the absence of the knowledge of the mechanistic insights through which the object has been created, the assembly pathway represents the amount of historical contingency (informational constraints along the construction pathway) required to construct the object. This means that in AT there are two experimental measurements that need to be made, the copy number and the AI. In this regard, AT cannot be said to reduce measurement between life and non-life to a scalar, as Hazen *et al*. claim the theory does. In principle, only objects with relatively high copy number (much greater than one) are experimentally observed, so implicitly, the experimentally measured AIs are always with high copy number. This is a subtle point and something it appears that Hazen *et al.* might have missed [1]. It is trivial to show that it is, in principle, possible to find an object produced abiotically with many parts, i.e. a high AI, but AT shows it is not possible to find many identical objects (high copy number) with a high AI produced abiotically. Assembly theory has been designed so that both the AI and abundance can be measured for organic molecules using mass spectrometry. The capacity to make real measurements is the crux of the ability to use AT as a life detection tool.

Assembly theory, as a theory, has a more fundamental application in showing how selection had to operate before biological selection. Hazen *et al*. misinterpret AT’s threshold concept, suggesting a universal threshold of AI greater than or equal to 15. However, this threshold applies specifically to covalent organic molecules based on experimental findings. What has been reported are experiments confirming the existence of a threshold, where for molecular assembly (e.g. AI of covalently bonded molecules), the threshold of AI greater than approximately 15 was identified when no abiotic samples tested exhibited a measured value above this threshold, but biological samples did [2–8]. The hypothesis of AT is that a threshold should exist, but the value of AI greater than approximately 15 for covalently bonded molecules was determined by experiment for those molecules with high enough abundance to be detectable. It is interesting to think about how complex inorganic molecules might be treated in AT, and indeed, one of us presented work on this [9] that pre-dates the paper of Hazen *et al.* [1]. Our previous work also explored metal oxides, and while heteropolyanions can be isolated in solution, they are very labile, forming a multitude of ion-pair oligomers [10]. In the literature on AT, a central tenet repeatedly stated is how to confirm validity for biosignature assessment, the AI must be corroborated by its experimental measurement [4–11] (e.g. [12], for a recent review). Given the vast number of equilibria available to heteropolyanions [3] and highly charged molecules in general, the AIs for inorganic non-covalent systems are probably not experimentally observable as it is not possible to determine the abundances of identical species because there is such a wide distribution.

The hypothesis explored by Hazen *et al.* [1] regarding a threshold value of AI for life detection in heteropolyanions is itself not currently testable. A clear example of where this can mislead the reader is in the authors’ suggestion that their calculations have been applied to ‘molecules’, while no covalently bonded structures are considered in the manuscript. There is an important and physically meaningful difference between measurable molecular structures and synthetic analogues to hypothesized mineral precursors. The claims of the Hazen *et al*. paper are based on the latter, where the calculations presented include only ‘molecule-like’ heteropolyanions. Comparing the computed values of AIs for these heteropolyanions to molecular assembly values of covalently bonded molecules is of limited utility since they are only superficially alike. In [8], we have used covalent bonds as building blocks. The literature makes clear how the AI can be applied to systems constructed from different sets of building blocks other than covalent bonds. Although the theory is general and can apply to different classes of substrates and materials made of different building blocks, it is still the case that one should only compare objects within the same class when discussing a threshold for life detection [7].

For example, the AI would be different if we chose atoms, quarks or some other structural motif instead of covalent bonds to build molecules, demonstrating how a threshold for life detection would be expected to be different as explained in [8]; however, covalent bonds are used because this is what is empirically testable using spectroscopic techniques [9,12] and is also consistent with the principles of AT that the steps in the assembly pathway should be physically implementable. Therefore, even if the hypothetical AIs of the inorganic structures discussed in Hazen *et al*. [1] were experimentally verifiable, they still could not be directly compared to molecular assembly values for organic molecules because the AI values are calculated in a different way, with different building blocks. The hypothesis of AT is that a threshold will exist, but there is no expectation it will be the same for all objects, independent of how they are constructed (what assembly space they live in; see figure 1) [13]. We should expect that if an AT of minerals can be developed and experimentally verified, it will have a different threshold for life detection than covalently bonded chemistry. This is one reason we agree with the authors of Hazen *et al*. that mineral biosignatures should be an exciting frontier for the application of AT (figure 2).

**Figure 1.**
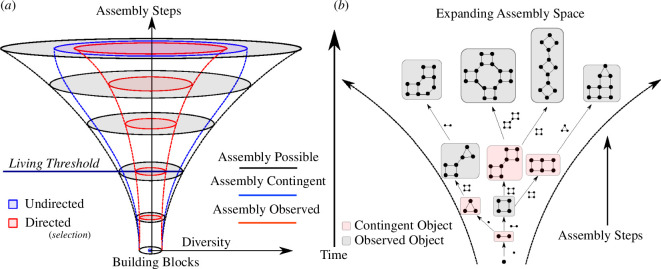
(*a*) A schematic showing the combinatorial size of space in AT as a function of the number of steps and diversity of building blocks. The assembly possible is the universe where the laws of physics and chemistry apply, but with no causal history. The assembly contingent is the same as the assembly possible but now with contingency required. The assembly observed is the universe of objects we actually see. (*b*) A depiction of how the assembly contingent expands in time and over assembly steps giving both contingent and observed objects.

**Figure 2.**
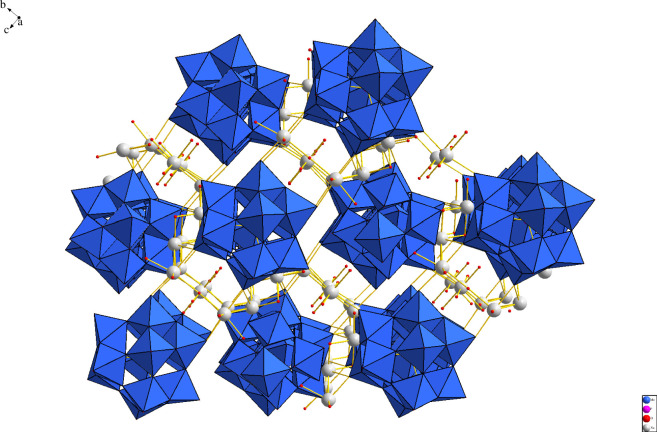
Drawing of a crystal structure of the {PMo_12_O_40_}^3−^ Keggin anion shown by the blue polyhedra with sodium ions connecting the anions together in a three-dimensional lattice. In solution, the Keggin ion forms a vast range of ion pairs [3].

Hazen *et al*. claim that an AI threshold of 15 does not serve as a reliable biosignature. However, this contradicts existing experimental evidence, which demonstrates that AIs greater than approximately 15 are only observed in systems associated with life or technology. It is possible the threshold might shift with more experimental data, but we have thus far seen no evidence to refine this.

We contend that claims of whether a given mineral, or molecule, can only form via an evolutionary process (is a sign of life) must be something that can be rigorously tested in the laboratory, so we regard it as premature to assign ‘sign of life’ to any structure—inorganic molecule, mineral lattice or otherwise—in the absence of strong empirical and theoretical grounding of the claim. To date, measurements of organic molecules with AI greater than approximately 15 have only been found experimentally to be produced from systems that are associated with life or technology [5–7], and this is motivated by a theoretical explanation [4].

We think that the study by Hazen *et al.* [1] raises interesting questions about the nature of chemical complexity, the limits of measurement of that complexity and the development of AT to encompass inorganic systems. In addressing this very interesting class of materials, we need to think critically about the measurement of ‘objects’, be they isolated molecules or extended lattices, and to consider what experimental techniques can be used to read out the shortest path of assembly. Mass spectrometry is particularly well suited to molecules, but we have recently extended this to infrared and NMR spectroscopy [4–9,11].

What is clear from the Hazen paper [1] is that AT is provoking people to think about the measure of complexity beyond standard information theory, which is subjective in nature as it needs an observer to assign the states. Assembly theory, in contrast, is a new quantitative way to follow and quantify selection and evolution. We believe the paper was a success in exploring these ideas, and we hope this comment is helpful in addressing the various misunderstandings and misrepresentations. This misunderstanding of Hazen *et al*. underscores the challenge in experimentally validating those theoretical AIs. This is because the scientific method is at its most powerful when theory and experiment work together, and suggesting examples that are not, in principle, testable will not advance the field. Misunderstanding that the calculation of the AI is the same as measuring it seems to be a fundamental error. A possible next step to correct this error might be for Hazen *et al*. to experimentally measure the AIs of the compounds they suggest have AIs greater than 15 and see if their analysis is corroborated by the experimental data. These results could be used to probe whether a threshold exists between abiotic and biologically produced inorganic molecules and minerals [11,12].

## Data Availability

This article has no additional data.
